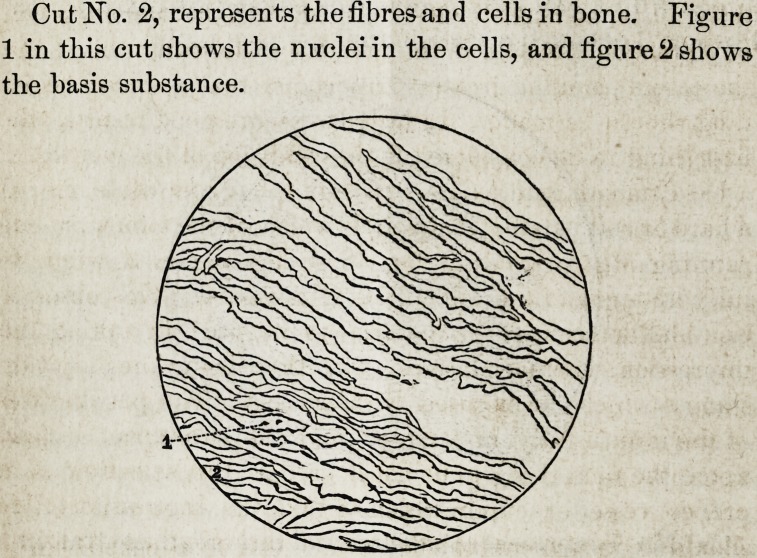# The Discovery of Cells with Fibres, Dentine at the Junction of the Enamel and Cementum

**Published:** 1870-05

**Authors:** Geo. B. Harriman

**Affiliations:** Boston.


### AMERICAN JOURNAL

OF

### DENTAL SCIENCE.

**Vol. IV. THIRD SERIES--MAY, 1870. No. 1.**

### ARTICLE I.

### *The Discovery of Cells with Fibres in Dentine at the Junction of the Enamel and Cementum.*

By Geo. B. Harriman, D. D. S., Boston.

In the Dental "Cosmos" for February of the present year, there is an article on the development of cells of the dentinal pulp into tubuli.

In his introductory, the author premises that "the effect of this tubular arrangement within the structure of the dentine is a matter which not only interests those who study Dentistry as an art, but that other class who are devoted to it as a science." With this utterance I am in entire sympathy and particularly with the concluding part, the consideration of Dentistry as a science. Scientifically considered, I must take exceptions to principles submitted in the article now under consideration.

What are we to understand by the term tubuli?

By Kolliker and almost every other writer on the subject "tubes and tubuli are hard pipes which admit of the passage of fluid." By the author of the article in question, they are likened to the "tubes of a boiler and hollow columns of a building."

My researches and long continued study, lead me *away* from such histology and physiology as this.

From repeated experiments, I am prepared to demonstrate and prove that these so styled "tubuli" are cells and fibres which radiate from the pulp cavity and terminate at the junction of the enamel and cementum. At this point these cells and fibres can be frequently seen as real soft solids, as much so as the fibres and cells in cartilage.

A careful examination with a microscope of medium high power, from four to seven hundred diameters, will soon convince us that no fluid passes in these so called "tubuli." What is seen are calcareous salts. As an illustration of this position, take a freshly extracted tooth extirpate its pulp, then inject it with carmine, and afterwards make thin sections longitudinally, crossing of course the so called "*tubuli*," but not interfering with the pulp cavity, and the experiment will show *no* entrance of carmine into these "pipes." Whereas had they been *hollow*, the carmine would have penetrated at once and have filled them.

It is to be presumed that every writer upon this subject has made the tooth a matter of profound study, and has summoned to this aid the wise and learned, who likewise have largely investigated and have given to the world the results of their investigations. It is to be borne in mind, however, that Dentistry as a science in just in its dawn, and that, consequently, past investigations were pursued in comparative darkness. This dawn is rapidly increasing in effulgence and chases back the clouds of the past, making light so clear and bright as to allow every *open* eye to behold the truth and welcome it to the heart and intellect. In these "tubuli" are seen real *cells* and *fibres*, nothing more nothing less. The interrogatory may now be put, what are the functions of these cells and fibres?

I answer their office is to build up the dentine of the tooth by the deposit of calcareous salts, which are their *only* contents.

According to Kolliker, these cells and fibres, as I denominate them or as they are otherwise called "tubuli" are about the ten thousandth of an inch, but select the scale of Bealing for measurement they are really about the five thousandth of an inch. When disencumbered, their measurement is from the thirty-five hundredth to the four thousandth of an inch, thus showing that they *swell* when released from surrounding substance. I have made a great number of very thin sections of the teeth, as thin as the three thousandth of an inch.

It may be interesting to the reader to be made acquainted with the process. These sections can be made of any desired size and attenuated to any point—from two hundredths to three thousandths of an inch. My process is to glue the tooth into a piece of wood, and fasten the wood in a lathe where there is a carriage which runs in a rack, or pinion with a tool post to guide the cutting instrument. In this way I obtained a section after my own desire.

Cut No. 1 represents the fibres and cells of a central incisor tooth; (1) in this cut shows the nuclei in the cells after application of diluted acetic acid; (2) shows cells closely impacted; (3) shows the interstitial substance or lime salts.

Placing one of them on a glass slide, with a microscope having a prism in the objective as well as reflected light from beneath the object, I have seen the dissolution of the hard substance, and the enlargement or *swell* of the cells and fibres, from about the five thousandth of an inch to the thirty-five hundredth of an inch in diametor. "Tubuli" are compared, in the article we are noticing, to the hollow tubes in long bone, woody fibres, etc.

From the preceeding remarks, the reader is prepared for my protest against such a theory. There are no "Tubuli" in woody fibres, nor is there any hollow tube in bone as I can clearly prove, and as cut 2. illustrates. Bone has cells and fibres which can be readily detected and exposed by making thin sections, after the manner already indicated above, the twenty-five hundredth or three thousandths of an inch in diameter, and employing hydrochloric acid to dissolve the hard substance; which experiment I would commend to all interested.

Cut No. 2, represents the fibres and cells in bone. Figure 1 in this cut shows the nuclei in the cells, and figure 2 shows the basis substance.

The soft solids within the cementum of the tooth, are analogous to those in bone, and are composed of cells and fibres, and there are no hollow tubes or canals in the cementum.

The matter presented in this article is of much importance, and cannot fail to interest every one engaged in the dental or medical profession. It may provoke thought, and excite to research and experiment, and thus more light may be obtained for the better understanding of this branch of science.

## Figures and Tables

**Cut No. 1 f1:**
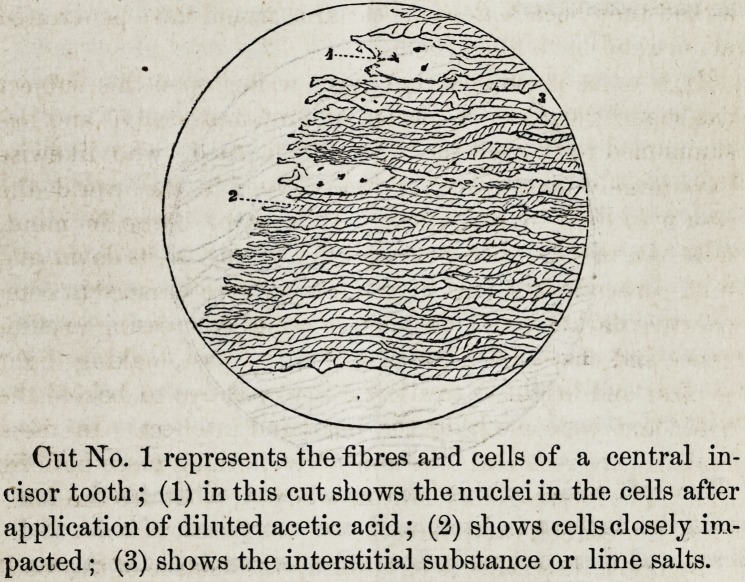


**Cut No. 2 f2:**